# Protein use efficiency and stability of baking quality in winter wheat based on the relation of loaf volume and grain protein content

**DOI:** 10.1007/s00122-022-04034-x

**Published:** 2022-01-28

**Authors:** Friedrich Laidig, Alexandra Hüsken, Dirk Rentel, Hans-Peter Piepho

**Affiliations:** 1 Institute of Crop Science, University of Hohenheim, Biostatistics Unit, Fruwirthstrasse 23, 70599 Stuttgart, Germany; 2 Department of Safety and Quality of Cereals, Max Rubner-Institut, Federal Research Institute of Nutrition and Food, Schuetzenberg 12, 32756 Detmold, Germany; 3 Bundessortenamt, Osterfelddamm 60, 30627 Hannover, Germany

## Abstract

**Key message:**

A novel approach based on the loaf volume–grain protein content relation is suggested to consider the static protein use efficiency and stability as efficient quality-related descriptors for wheat varieties.

**Abstract:**

The most important trait for baking quality of winter wheat is loaf volume (V). It is mostly determined by grain protein content (GPC) and quality. New varieties with a high potential of grain protein use efficiency (ProtUE) are very important for reducing the surplus use of nitrogen fertilizer in areas where nitrogen leaching is large. This is also an important goal of agricultural policies in the European Union. Additionally, ProtUE needs to be very stable across environments in the face of progressing climate change with more volatile growing conditions. We evaluated a new approach to assess ProtUE and stability based on the V–GPC relationship instead of using only single traits. The study comprised 11,775 baking tests from 355 varieties grown 1988–2019 in 668 different environments in Germany. V was predicted by quadratic and linear regression functions for quality groups, indicating a reduction of ProtUE from 1988 to 2019. We introduced a dynamic and a static approach to assess ProtUE and stability as potential criteria in variety registration. We found a considerably lower heritability of the dynamic ProtUE (*h*^2^ = 43%) compared to the static ProtUE (*h*^2^ = 92%) and a lower dynamic stability (*h*^2^ = 32%) than for the static stability (*h*^2^ = 51%). None of these measures is in conflict with the selection for high V. In particular, V and static ProtUE are strongly genetically associated (*r* = 0.81), indicating an advantage of the static over the dynamic approach.

**Supplementary Information:**

The online version contains supplementary material available at 10.1007/s00122-022-04034-x.

## Introduction

Baking quality of winter wheat is mostly determined by grain protein content (GPC) and quality, where loaf volume (V) is the most important quality-determining trait. GPC is widely used in evaluating V and a higher price is usually achieved with high-protein bread wheat. However, many studies have already demonstrated that large differences exist in the proportion of the variation in V that can be explained by the variation in GPC (Hasniza et al. 2014; Johansson and Svensson 1998; Koppel and Ingver 2010). This was also shown for some registered varieties in Germany, which do not have a linear dependency between protein quantity and V, see, e.g., Gabriel et al. (2017). Those varieties have relatively low GPC; however, because of the high functionality of the gluten proteins, the baking performance of such wheat varieties is much better than expected from classical measurement results (Lindhauer 2014). Wieser and Seilmeier (1998) found for 13 German winter wheat varieties that the ratio of gliadin to glutenin changed in the same direction as the level of N fertilization, but the degree to which the ratio changed was variety-specific. Seling (2010) used samples from a 3-year variety registration trial containing 5 varieties with a range in GPC of 11–15%. In this study, 2 varieties showed no correlation of V with increased GPC (*r* = 0.10 and *r* = 0.14), whereas 3 varieties showed a positive linear relationship (*r* = 0.87 and *r* = 0.88). Furthermore, Borghi et al. (1999) could show that for some varieties a curvilinear instead of a linear increase in GPC exists in response to nitrogen fertilizers. Gabriel et al. (2017) found that the response of V to increasing GPC differed among varieties in shape and slope, showing that simply measuring GPC is partly insufficient in evaluating baking quality.

Until now, the continued improvement of baking quality is mostly a secondary objective in many bread wheat breeding programs, and an indirect selection for baking quality traits is often conducted, using relatively cheap and high-throughput tests like GPC via NIRS as a major selection criterion in early generations (Michel et al. 2019). A more future-oriented approach is to use the described differences between varieties to transform GPC into V in breeding programs and variety testing in order to identify varieties with high transformation potential, as protein use efficient varieties can contribute to enable sustainable, environmentally compatible and profitable bread wheat production. This is not only important with respect to the ongoing climate change but also in view of the goal of agricultural policies in the European Union, including Germany, to reduce the surplus of nitrogen fertilizer by about 20% in certain areas where nitrogen leaching is large. In addition, the production of nitrogen fertilizers is a very energy-intensive process and losses due to denitrification and volatilization and immobilization can also be permanent or temporary detrimental to the environment. Against the background of the required reduction of nitrogen surplus and hence nitrogen fertilizing rates, GPC in wheat varieties may be decreasing in the future (BMEL 2020; EU 2019). Thus, the increased availability of wheat varieties with improved protein functionality and baking quality at lower GPC in the future will require the identification and exploitation of additional efficient quality-related descriptors.

In general, modern winter wheat varieties are expected to show only moderate variability in baking quality under differing pedo-climatic conditions within and across years. Additionally, stability of grain quality is important for the milling and baking industry, since it guarantees consistent procedures and reduces product loss during processing, especially when switching from 'old' to 'new' stocks in the months following harvest. Although the importance of quality stability is unquestionable (Johansson et al. 2020), there is no consensus how to define or measure it. To evaluate the stability of baking quality traits, two different approaches are possible, the static or the dynamic concept, both based on single quality traits. The concept of static stability considers a cultivar as stable if its performance does not change across environments (Becker and Leon 1988), whereas according to the dynamic concept of stability, a cultivar is considered as stable if its performance follows the given potential of an environment, often expressed as the average performance of all grown cultivars in the respective environment (Becker and Leon 1988). Many stability measures have been introduced that can be associated with one of the mentioned concepts (Lin et al. 1986). Most stability analyses focus exclusively on yield whereas few have considered the stability of baking quality. Static stability is recommended for characterizing quality traits (Becker and Leon 1988) because it corresponds best to the need for a constant or at least regular quality. Knapp et al. (2017) compared stability measures of both concepts for most important traits of baking quality in winter wheat. Results showed that no trade-off between the two stability concepts exists and the development of cultivars that are of both stable and of high quality should be possible.

Against this background, we introduce in this study a new approach to describe protein use efficiency and stability of baking quality, not with respect to a single trait of baking quality, but based on the relation of V and GPC (Hüsken 2020). We define protein use efficiency (ProtUE) for wheat varieties as loaf volume (V) divided by the amount of grain protein content (GPC). Physiologically, ProtUE indicates the grain protein composition and the resulting ability to produce V as a function of GPC. Highest ProtUE will be obtained if the protein quality is high and GPC is kept to a minimum. (V is measured as the volume based on 100 g flour; for brevity, the reference amount of 100 g will not be mentioned in the following.) In this study, we use the V–GPC relation to define the ProtUE as the ratio of V/ GPC and consider two approaches for its assessment, a dynamic ProtUE and a static ProtUE. The dynamic ProtUE expresses the regression of V on GPC. The static ProtUE will be given by the sample average of the ratio V/GPC (ml/% GPC).

The aim of this paper is (i) to describe the quality group-specific V–GPC relation over all varieties by a mixed linear model, (ii) to evaluate variety-specific stability measures of the V–GPC relation according to the dynamic and static approaches, (iii) to compare the predictive power of ProtUE and stability measures by estimating their heritability, and (vi) to consider whether they may be suitable as efficient quality-related descriptors of winter wheat varieties.

## Materials and methods

### Data set

This study was based on data from official variety trials for winter wheat carried out across Germany from 1988 to 2019. Newly bred candidate varieties in Germany have to be evaluated for their value of cultivation and use (VCU) before they can be registered to the National List (NL) and released for commercial production. Besides yield and disease resistance, quality traits are important performance traits. The regular testing period for a candidate variety lasts 3 years. Each year 3 trial series were conducted, where varieties in their first testing year were grown in trial series S1, in their second in S2 and in the last in S3, hence a testing cycle covered trial series S1, S2 and S3, e.g., a variety with first testing year 2000 is tested in S1 in 2000, in S2 in 2001, and in S3 in 2002. In the following. we denote the 3-year testing period as “testing cycle” or simply “cycle” and indicate the cycle in which a variety was tested by its first testing year, e.g., “cycle 2002” refers to the testing period 2002–2004. Additionally to the candidate varieties, at least 3 reference varieties were included in each series, which were identical over sites and temporal series of the same cycle. Well-established varieties were chosen as references, representing the actual state of breeding progress. The references were updated on a regular basis, ensuring at least partial overlap of sets of references used in successive years. The number of locations in each trial series was in the range of 14 to 25. For the assessment of baking quality, bulked samples from 2 replications were drawn for each variety using 8 locations in each trial series. In total 24 samples were available for quality assessment for each variety over its 3-year testing cycle. The samples were drawn from the treatment intensity receiving nitrogen fertilizer, fungicides, herbicides and plant growth regulators according to good agronomic local practice. Nitrogen fertilization rate did not substantially change between 1988 and 2019, and so was not included as a covariate.

The German wheat classification system grades varieties according to their baking quality as part of the registration process. C-grade varieties have the lowest quality, followed by B-grade (bread making), A-grade (quality) and E-grade (elite) varieties having the highest quality grade. Allocation of a variety to one of these quality groups was dependent on particular minimum requirements with respect to individual quality traits (Bundessortenamt 2021, p. 154 ff.).

The data set contained 11,775 samples from 355 released varieties, 43 of which were references. Reference varieties were run in trials for about 7 years. In total 668 trials were grown in the period 1988 to 2019 comprising 30 overlapping testing cycles. 13% of the samples were from C-grade, 36% from B-grade, 37% from A-grade and 14% from E-grade varieties.

### Laboratory tests

V (ml) was assessed by a standard baking test based on the Rapid-Mix-Test (RMT) and measures the volume of bread rolls obtained from 100 g flour of type 550 (Arbeitsgemeinschaft Getreideforschung e.V. 2016, pp. 97–104). The flour was milled according to the standard milling test (Arbeitsgemeinschaft Getreideforschung e.V. 2016, pp. 33–36). Prior to dough preparation, the moisture content of the flour was determined with an Inframatic 8600 (Perten Instruments GmbH, Hamburg, Germany) according to DIN EN 15,948:2012, falling number was measured according to Hagberg-Perten DIN EN ISO 3093:2009, and water absorption (ml/100 g) was determined using a farinograph according to ICC 115/1. GPC of the coarse meal was measured using the standard method ICC 105/2 and DIN EN 15948.

## Statistical analysis

### Basic model for estimation of variance components and genotypic correlation

For a given observation, we used the linear mixed model with factors genotype G, location L and year Y and trial series T and considering genetic and non-genetic long-term trends given by

1 $$\begin{aligned} y_{ijkl} & = \mu + \beta r_{i} + \gamma t_{j} + G_{i} + Y_{j } + L_{k} + \left( {YL} \right)_{jk} + \left( {YLT} \right)_{jkl} \\ &\quad + \left( {GL} \right)_{ik} + \left( {GY} \right)_{ij} + e_{ijkl} \end{aligned}$$

where *y*_*ijkl*_ is the trait mean (V, GPC, V/GPC) of the *i*th genotype in the *j*th year, *k*th location and the *l*th trial series within year and *μ* is the intercept, *β* a fixed regression coefficient for the genetic trend, *r*_*i*_ the first year in trial of *i*th genotype, *γ* a fixed regression coefficient for the non-genetic trend, *t*_*k*_ the covariate for the *j*th calendar year, *G*_*i*_ the main effect of the *i*th genotype, *Y*_*j*_ the main effect of the *j*th year, *L*_*k*_ the main effect of the *k*th location, (*YL*)_*jk*_ the *jk*th location × year interaction effect, *(YLT)*_*jkl*_ the effect of trial series within year × location interaction, (*GL*)_*ij*_ the *ij*th genotype × location interaction effect, (*GY*)_*ik*_ the *ik*th genotype × year interaction effect, and $e_{ijkl}$ a residual effect comprising both genotype × location × year interaction and the error of a mean arising from sampling the replications. All effects except *μ*, *β* and *γ* are assumed to be random and independent with constant variance for each effect.

### Model for predicting relation of V to GPC

To visualize the V–GPC relation over all varieties considering quality groups, we used GPC as covariate and quality groups as categorical effects in Eq. (1). We allowed for linear and quadratic terms in GPC which we centered at its mean value. The result of the model selection process (see Hadasch et al. 2020) is given by

2 $$E\left( {V_{ijklm} } \right) = \alpha_{m} + \beta_{m} t_{j} + \gamma_{m} GPC_{ijkl} + \delta_{m} GPC_{ijkl}^{2}$$

where *E*(*V*_*ijklm*_) is the expected value of $V_{ijklm}$ of the *i*th genotype within the *m*th quality group in the *j*th year, *k*th location and the *l*th trial series within year and *μ* is the intercept, $\alpha_{m}$ is the categorical effect of baking quality of *m*th group, $t_{j}$ is the covariate for the *j*th year, $GPC_{ijklm}$ is the covariate for GPC and $\beta_{m}$, $\gamma_{m}$, $\delta_{m}$, are the corresponding regression coefficients of *m*th quality group. The estimates of Eq. (2) are given in Table 1.

**Table 1:** Regression model for prediction of loaf volume (V) by grain protein content (GPC) for 1988–2019 (Eq. (2))

Coeff		Estimate	SE	*t*	*P*(*t*)
$\alpha_{m}$	C	556.750			
	B	611.850			
	A	649.070			
	E	676.700			
$\beta_{m}$	C	−1.816	0.449	4.1	< .0001
	B	−1.885	0.371	−5.1	< .0001
	A	−2.002	0.362	−5.5	< .0001
	E	−2.009	0.398	−5.1	< .0001
$\gamma_{m}$	C	9.482	0.995	9.5	< .0001
	B	15.205	0.616	4.7	< .0001
	A	19.053	0.594	32.1	< .0001
	E	25.311	0.951	26.6	< .0001
$\delta_{m}$	C	−0.879	0.525	−1.7	0.0938
	B	−1.178	0.325	−3.6	0.0003
	A	−1.358	0.310	−4.4	< .0001
	E	−0.945	0.339	−2.8	0.0053

*Coeff* regression coefficients [Eq. (2)]; *Estimate* estimates of regression coefficients; *SE* standard error; *t t*-value; *P(t)* p-value of *t*-value

### Dynamic ProtUE

Stability analyses were based on GPC and V and done separately for each variety. The dataset available for each variety comprised data from a complete testing cycle (trial series S1, S2, S3) and 8 locations in each series, with some possible overlap of locations in S1, S2 and S3. The total number of observations available per variety was 3 × 8 = 24. In the following, we do not use subscripts for variety, but it is understood that any equation given is assessed per variety. Equations will only have a subscript *j* for year and *k* for locations.

Stability for V is assessed using a regression on GPC. The basic regression model is

3 $$V_{jk} = \mu_{V} + \beta_{D} GPC_{jk} + e_{jk} ,$$

where $\mu_{V}$ is the intercept, $\beta_{D}$ is the slope and $GPC_{jk}$ is covariate for GPC. Throughout the paper, we mean-centered $GPC_{jk}$ so that the intercept $\mu_{V}$ became the mean of GPC. The slope $\beta_{D}$ is referred to as dynamic ProtUE, indicated by the subscript *D*. By comparison, we added the subscript *V* on the intercept $\mu_{V}$ to indicate that this refers to the response variable V.

To account for year-specific intercepts, Eq. (3) can be extended to

4 $$V_{jk} = \mu_{V,j} + \beta_{D} GPC_{jk} + e_{jk} .$$

A stability measure associated with this model is the standard deviation of deviations $e_{jk}$ from the regression, denoted as $\sigma_{D}$.

Furthermore, locations may overlap, and then Eq. (4) can be extended by a location main effect $L_{k}$ as

5 $$V_{jk} =\mu_{V,j} + \beta_{D} GPC_{jk} + L_{k} + e_{jk} .$$

Here, the dynamic stability measure is given by

6 $$\sigma_{D} = \sqrt {\sigma_{L}^{2} + \sigma_{e}^{2} } ,$$

where $\sigma_{L}^{2}$ is the variance of $L_{k}$ and $\sigma_{e}^{2}$ the variance of $e_{jk}$.

Finally, to assess heterogeneity of slopes between years, we may also allow the slope to be year-specific:

7 $$V_{jk} =\mu_{V,j} + \beta_{D,j} GPC_{jk} + L_{k} + e_{jk} .$$

We tested for heterogeneity among slopes by using $\beta_{D,j} GPC_{jk} = \left( {\beta_{D} + \delta_{j} } \right)GPC_{jk} = \beta_{D} GPC_{jk} + \delta_{j} GPC_{jk}$, where $\delta_{j} GPC_{jk}$ is the interaction between year and GPC. If the interaction is not significant, homogeneity can be assumed. As heterogeneity was found to be significant in only few cases, we did not consider Eq. (7) further. Further, inspection of results obtained by Eq. (5) showed that for each cycle only a few locations were overlapping in trial year 2 (S2) and 3 (S3), while in trial year 1 (S1) at most one location was in common with year 2 and 3. This strong non-orthogonality of locations and years resulted in zero estimates of variance components for the location random effect *L*_*j*_. Therefore, subsequent analyses were done by using Eq. (4) assuming a common slope $\beta_{D}$ but allowing for year-wise intercepts $_{V,j}$.

Subsequently, the estimators $\beta_{D}$ and $\sigma_{D}$ of Eq. (4) are denoted as the dynamic ProtUE *b*_*D*_ and the dynamic stability *s*_*D*_, respectively.

### Static ProtUE

Before considering the static ProtUE, we examine the properties of the ratio V/GPC. If we assume that V and GPC are correlated variables, then the expected value and variance of V/GPC can be approximated by the means, variances and covariance of V and GPC using the delta-method as described, e.g., by Johnson et al. (1993, p. 55). Hence, the expected value of V/GPC is given by

8 $$E\left( {V/GPC} \right) = \mu_{V/GPC}\approx \frac{{\mu_{V} }}{{ \mu_{GPC} }}\left[ {1 + \frac{{\sigma_{GPC}^{2} }}{{\mu_{GPC}^{2} }} - \frac{{\sigma_{V, GPC} }}{{\mu_{V} \mu_{GPC} }}} \right],$$

and the variance by

9 $${\text{var}} \left( {V/GPC} \right) = \sigma_{V/GPC}^{2} \approx \frac{{\mu_{V}^{2} }}{{\mu_{GPC}^{2} }}\left[ {\frac{{\sigma_{V}^{2} }}{{\mu_{V}^{2} }} - \frac{{2\sigma_{V,GPC} }}{{\mu_{V} \mu_{GPC} }} + \frac{{\sigma_{GPC}^{2} }}{{\mu_{GPC}^{2} }}} \right].$$

It should be noted that the expected value of V/GPC is not the same as the ratio of the expected values of V and GPC; in fact, Eq. (8) shows that the expected value V/GPC depends on the squared coefficient of variation for GPC, where $CV_{GPC} = \frac{{\sigma_{GPC} }}{{\mu_{GPC} }}$ and on $CCV_{V GPC} = \frac{{\sigma_{V, GPC} }}{{\mu_{V} \mu_{GPC} }}$, which we denote as the coefficient of the covariation (CCV) between V and GPC. The larger the $CCV_{V GPC}$, the smaller is the bias. Thus, the expected value of V/GPC is closer to the ratio of their means, when there is a strong positive correlation. The variance of V/GPC is approximately equal to the ratio of the squared means of V and GPC, multiplied by a factor which depends on the sum of the squared CV for V and GPC minus twice the CCV. This means that the variance of V/GPC gets smaller if both variables are positively correlated, but increases if they are negatively correlated.

Stability of V/GPC is assessed by the mean and variance over environments. Hence, the linear model is given by

10 $$V/GPC_{jk} = \mu_{V/GPC} + f_{jk} ,$$

where $\mu_{V/GPC}$ is the expected value of V/GPC, denoted as the static ProtUE, and $f_{jk}$ is a random deviation of environments from $\mu_{V/GPC} .$

Further, the mean $\mu_{V/GPC}$ can be assumed to be year-specific, then Eq. (10) is changed to

11 $$V/GPC_{jk} = \mu_{V/GPC,j} + g_{jk} ,$$

where $\mu_{V/GPC,j}$ is the expected value of V/GPC of the *j*th year and $g_{jk}$ is the random deviation from the expected value $\mu_{V/GPC,j}$ in the *jk*th environment. $g_{jk}$ is assumed to have standard deviation $\sigma_{V/GPC}$, which is our static stability measure for V/GPC.

The estimators of $\mu_{V/GPC}$ given by Eq. (10) and $\sigma_{V/GPC}$ given by Eq. (11) are subsequently denoted as the estimators of static ProtUE *m*_*S*_ and static stability *s*_*S*_, respectively.

### Heritability and genotypic correlation of dynamic and static ProtUE measures

Heritability of all measures was estimated by

12 $$z_{ij} = \mu + \beta r_{i} + \gamma c_{j} + G_{i} + C_{j} + e_{ij} ,$$

where $z_{ij}$ is the measure of interest (mean of V, *m*_*V*_; mean of GPC, *m*_*GPC*_; dynamic ProtUE, *b*_*D*_; dynamic stability, *s*_*D*_; static ProtUE, *m*_*S*_; or static stability, *s*_*S*_) of the *i*th variety in the *j*th cycle, $\mu$ is the overall mean, β is a fixed regression coefficient for the genetic trend, *r*_*i*_ is the first year in trial of the *i*th genotype, γ is a fixed regression coefficient for the non-genetic trend, *c*_*j*_ is the covariate for the *j*th cycle year, *G*_*i*_ is the effect of *i*th variety, *C*_*j*_ is the effect of *j*th cycle year, and $e_{ij}$ is the residual effect which comprises the residual error and the G × C interaction effect. If the effects *G*_*i*_, *C*_*j*_ and *e*_*ij*_ are considered as random with variance $\sigma_{G}^{2} , \sigma_{C}^{2}$ and $\sigma_{e}^{2}$, respectively, then the heritability of cycle-based measures is given by

13 $$h^{2} = { 1}00\% \frac{{\sigma_{G}^{2} }}{{\left( {\sigma_{G}^{2 } + \sigma_{e}^{2} } \right)}}.$$

Further, genotypic correlation coefficients between measures *z*_*ij*_, as given by Eq. (12), were estimated. The correlation between two random effects in Eq. (12) can be considered as the association between two measures solely due to this random effect. For example, the correlation of genotype effects *G*_*i*_ for *b*_*D*_ and *s*_*D*_ is the genotypic correlation between the dynamic ProtUE and its stability. A genotypic correlation coefficient of *r* = 0 indicates that the genotype effects between measures were independent and with *r* = 1 they were entirely associated. We used a univariate approach from which correlation coefficients for random effects can be inferred. The method is based on Piepho et al. (2014) and described in Supplementary Material SM.

### Standard error for dynamic and static ProtUE measures

The standard error *se*_*z*_ of the estimated measures *z* (Eq. (12)) is an important indicator for their accuracy. However, the estimated measure *z* for a new variety in a new cycle is larger than the estimated sample standard error *se*_*z*_. Due to the presence of both G × C interaction and the residual error (squared standard error of the estimate of *z*_*ij*_) in the residual effect *e*_*ij*_, the estimate for a single cycle confounds the main effect *G*_i_, our target here, with the residual effect *e*_*ij*_. This means that the reported standard error *se*_z_ cannot be taken as face value, but must be inflated to reflect the presence of G × C interaction. Thus, for analyses of individual cycles for new varieties, we propose to report the following approximate standard error:

14 $$\widetilde{se}_{z} = \sqrt {\hat{\sigma }_{e}^{2} } ,$$

where $\hat{\sigma }_{e}^{2}$ represents the estimated residual variance of the error term *e*_*ij*_ given by Eq. (12).

## Results

### Genotypic and environmental variation and correlation

**Fig. 1:**
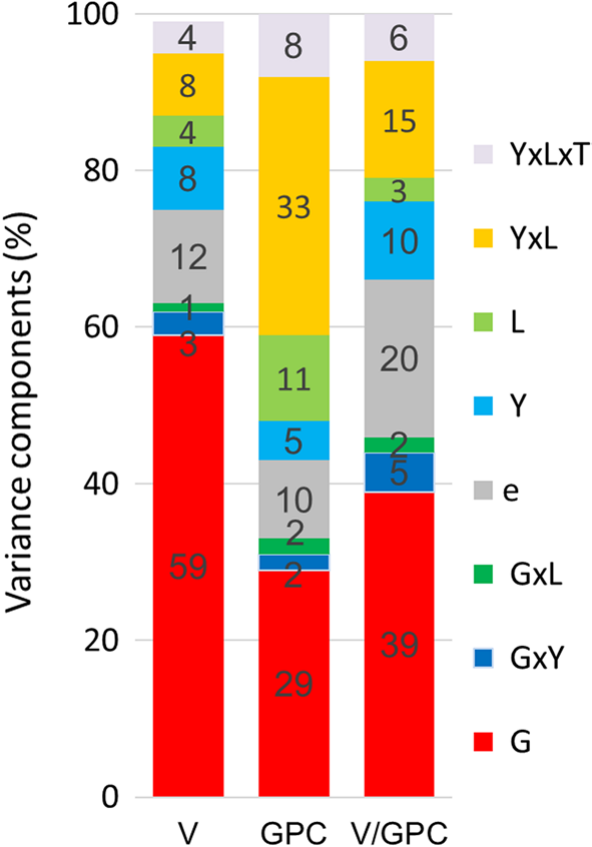
Variance components for loaf volume (V), grain protein content (GPC) and ratio (V/GPC) using Eq. (1). *G* Genotype; *Y* Year; *L* Location; *T* trial series; *e* Residual;

Baking quality, like other traits, is influenced by environmental conditions. Breeding for improved quality, change of crop management and climatic conditions may introduce long-term trends resulting in biased estimates for genotypic and environmental variation. Therefore, we considered genetic and non-genetic trends in Eq. (1) to avoid biased estimates of variance components. In Fig. 1 we show variance components of V, GPC and V/GPC expressed as percentage of their total sum to make the components comparable between traits. Genotypic variation was by far the largest component with 59% for V, 29% for GPC and 39% for V/GPC, followed by the interaction Y × L. For GPC, the variation of L (11%) and its interaction with years L × Y (33%) was considerably larger than the corresponding components for V, with 4% and 8% and for V/GPC with 3% and 15%, respectively. Variation of the residual effect *e*_*ij*_ for V/GPC (20%) was about twice as large as for V and GPC. The G × Y and G × L components were of minor magnitude, ranging from 1 to 5%. In general, GPC was subject to the largest environmental variation mainly caused by L and Y × L.

**Fig. 2:**
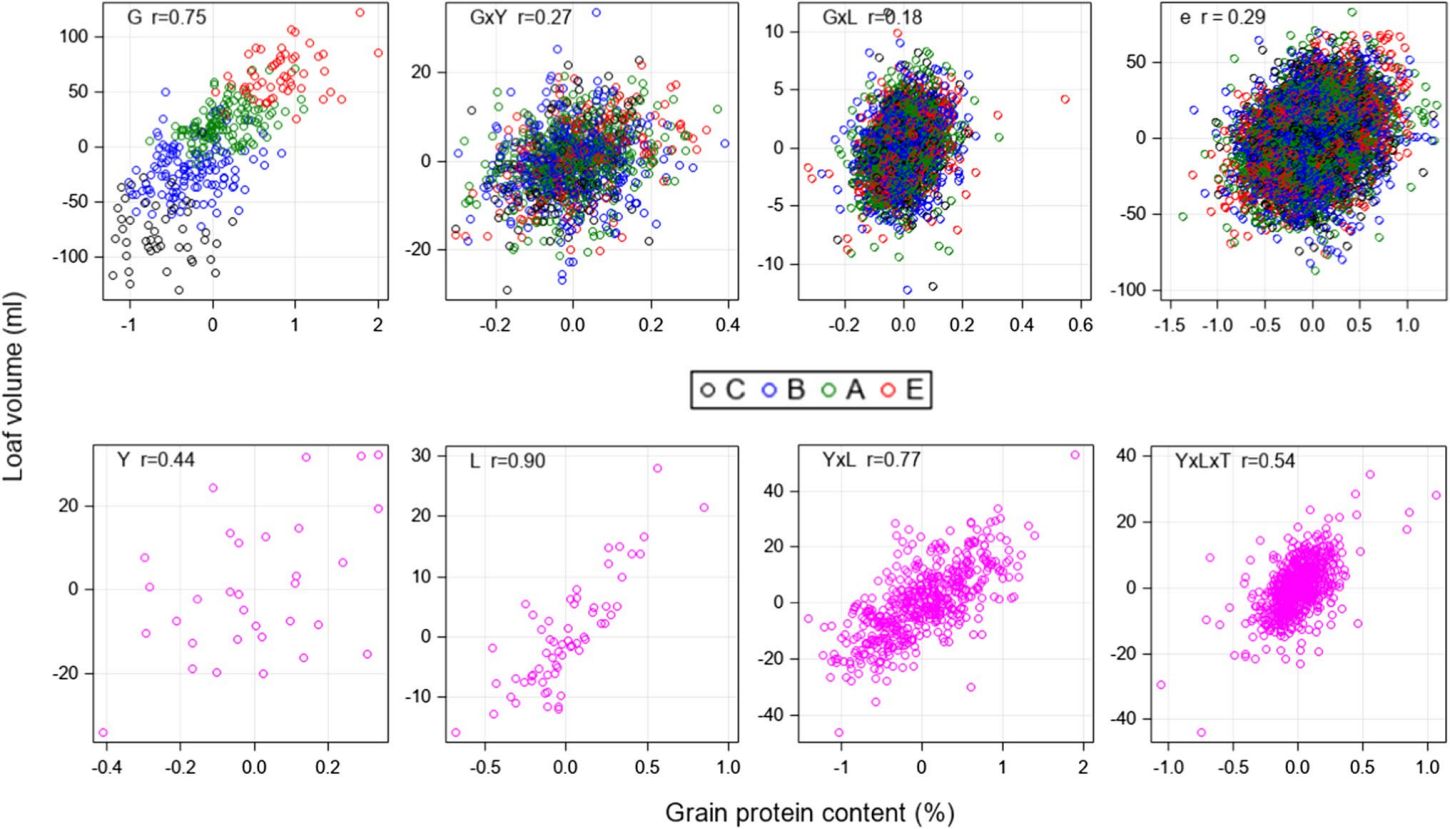
Correlation plots of estimated random effects and their correlation coefficients for loaf volume (*y*-axis) and grain protein content (*x*-axis) using BLUP (Eq. (1)). Note the plotted variables represent bivariate distributions of random effects with zero means. Plots in the upper row represent random effects for G, G × Y, G × L and e, colored by quality grades of varieties. Plots below represent the environmental random effects which are identical for all quality grades. *G* Genotype; *Y* Year; *L* Location; *T* trial series; *e* Residual; *C, B, A, E* Grade of baking quality

In Fig. 2 the correlation of V with GPC is shown based on the BLUP of the genotype, genotype × environment and environmental random effects as given by Eq. (1). The genotypic correlation showed a strong association with *r* = 0.75, with a clear separation of quality groups, while for the genotype × environment interaction the correlation was low with about the same dispersion for quality groups C, B, A and E. Contrary to the genotype × environment correlations, the effects of V and GPC are very strongly related for L (*r* = 0.90), strongly for the interaction Y × L (*r* = 0.77) and moderately for Y and for the trial series T (S1, S2, S3) within Y × L effects.

### Prediction of V by GPC

**Fig. 3:**
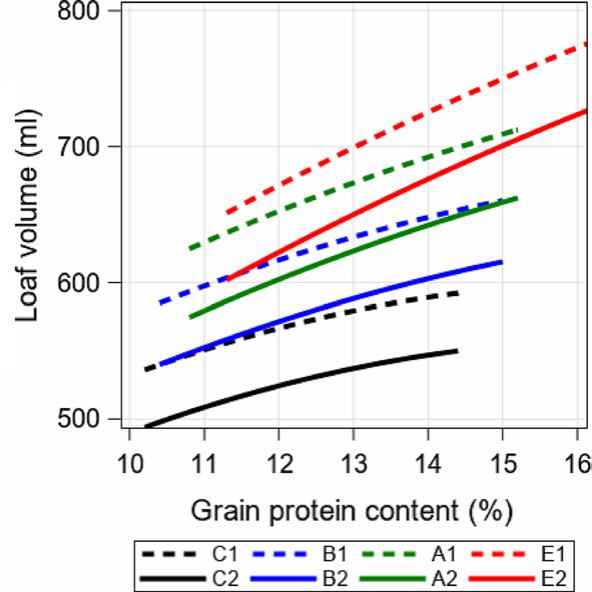
Loaf volume (V) predicted by Eq. (2) for individual quality groups for years 1990 (C1, B1, A1, E1, dashed lines) and 2015 (C2, B2, A2, E2, solid lines) plotted against grain protein content (GPC). The range of GPC corresponds to the 1% and 99% group-wise quantiles. *C, B, A, E* Quality grades of varieties (increasing quality from C to E)

The estimated regression coefficients are shown in Table 1 and the predicted V in Fig. 3 for year 1990 (dashed line) and 2015 (solid line) indicating a decrease in V-levels from 1990 to 2015 for varieties in all quality groups. For example, when considering A-grade varieties with GPC = 13%, then about 675 ml V were achieved in 1990, while in 2015 only about 623 ml were achieved. This corresponds to a reduction of 53 ml within 25 years for A-grade, 47 ml for B-grade, 46 ml for C-grade and 50 ml for E-grade varieties. However, the curves of the predicted V show a decreasing nonlinear steepness with increasing GPC. For group C to E, the steepness is decreasing with increasing GPC-values (Fig. 3), which is also indicated by negative quadratic regression coefficients as shown in Table 1.

### Examples for dynamic and static protein use efficiency (ProtUE)

**Fig. 4:**
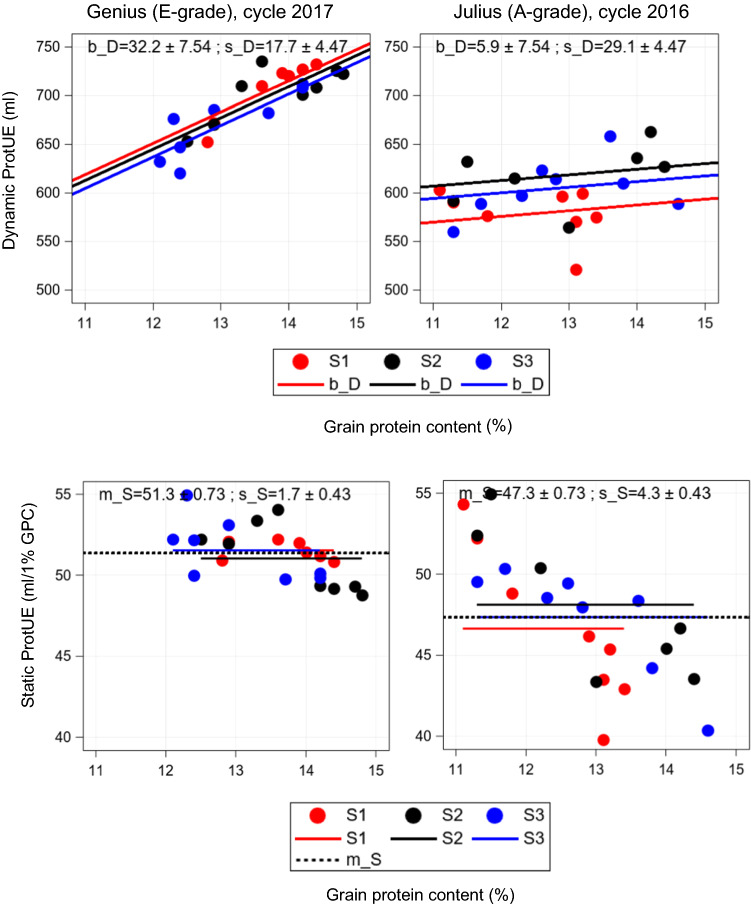
Examples demonstrating the dynamic ProtUE and dynamic stability (Eq. 4), the static ProtUE (Eq. 10) and static stability (Eq. 11) for variety Genius and Julius with quality grade E and A, respectively. Red, black and blue circles and lines represent environments from first (S1), second (S2) and third (S3) testing year, respectively. The dotted horizontal line represents the static ProtUE m_S. The estimated parameters and their standard errors (Eq. (14)) are given as insets. *b_D* Dynamic ProtUE (*b*_*D*_); *s_D* Dynamic stability (*s*_*D*_); *m_S* Static ProtUE (m_S_); *s_S* Static stability (*s*_*S*_)

The difference of dynamic and static ProtUE and their corresponding measures including standard errors are depicted in Fig. 4 for variety Genius (E-grade) in cycle 2017 and Julius (A-grade) in cycle 2016. Genius with dynamic ProtUE of *b*_*D*_ = 32.2 ml/1% GPC means that V gains 32.2 ml per 100 g flour by an increase of 1% GPC across environments in cycle 2017. We allowed for different intercepts per year but identical slopes. Therefore, parallel year-wise regression lines are shown. Observations of all 3 testing years showed low deviations from regression lines, indicating a high stability of dynamic ProtUE of *s*_*D*_ = 17.7. Compared to Genius, Julius had a lower dynamic ProtUE of *b*_*D*_ = 5.9 ml/1% GPC. Further, year-specific V levels differed stronger and deviations from the regression lines are larger than for Genius indicating a lower dynamic stability of *s*_*D*_ = 29.1.

The static approach of ProtUE was based on the cycle average *m*_*S*_ of V/GPC. The cycle average (black line) represents the static ProtUE which was higher for Genius with *m*_*S*_ = 51.3 ml/1% GPC than for Julius with *m*_*S*_ = 47.3 ml/1% GPC. This means that Genius was able to produce 51.3 ml and Julius 47.3 ml V per 1% GPC in cycle 2017 and 2016, respectively. We should note that the static stability measures the deviations of the observations from year-specific means. Variability of environments showed much larger deviations for Julius than for Genius indicating a higher static stability for Genius (*s*_*S*_ = 1.7) than for Julius (*s*_*S*_ = 4.3). Contrasting Genius and Julius suggests that a higher dynamic ProtUE *b*_*D*_ resulted in a higher static ProtUE *m*_*S*_ for Genius, but a lower *b*_*D*_ seems to be associated with a lower *m*_*S*_ as became apparent for Julius.

### Variance components and heritability of dynamic and static protein use efficiency measures

To reiterate, a candidate variety was regularly tested for 3 years in 8 environments (locations) each year, which we considered as one testing cycle. If measures of ProtUE and stability are to be used as reliable characteristics for registration, they should have a high predictive power, i.e., measures should be repeatable. We applied the heritability coefficient *h*^2^ (Eq. (13)) to describe repeatability of ProtUE measures. In the data set used, only varieties with at least 15 environments per cycle were included. In total 471 variety × cycle measures from 327 varieties over 30 cycles (1988–2017) were available. Thereof 32 varieties with at least 2 testing cycles were included with an average of 5.5 cycles per variety. The cycle means of V–GPC relation defining traits *m*_*V*_ and *m*_*GPC*_ were also included as a benchmark.

**Fig. 5:**
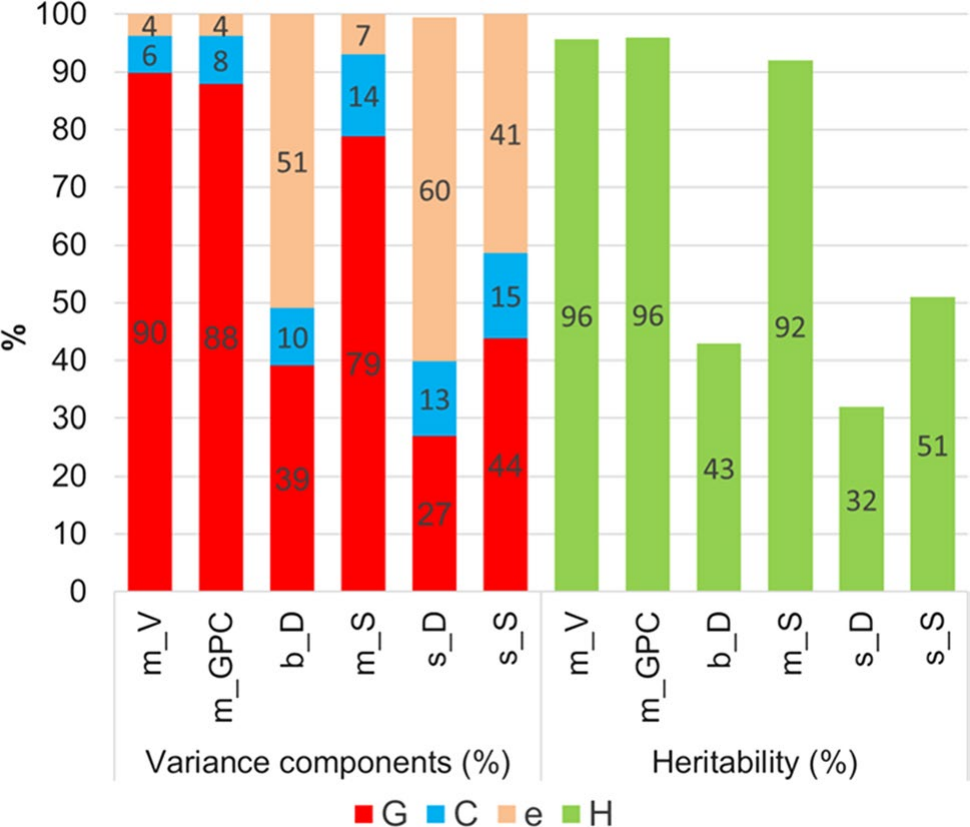
Variance components (Eq. (12)) and heritability (Eq. (13)) of dynamic and static ProtUE and stability based on variety × cycle measures. *m_V* Cycle mean of loaf volume (V); *m_GPC* Cycle mean of grain protein content; *b_D* Dynamic ProtUE (*b*_*D*_); *s_D* Dynamic stability (*s*_*D*_); *m_S* Static ProtUE (*m*_*S*_); *s_S* Static stability (*s*_*S*_); *G* Genotype; *C* Cycle; *e* Residual error

In Fig. 5, we show the relative magnitude of variance components as a percentage of total variance. The genotypic variation G was highest for the cycle means *m*_*V*_ (90%) and *m*_*GPC*_ (88%) and lowest for the dynamic stability measure *s*_*D*_ (27%). Variation of testing cycles C was low in the range between 6% for *m*_*V*_ and 15% for static stability *s*_*S*_. The residual variance for *e*_*ij*_ was very low for *m*_*V*_ (4%), *m*_*GPC*_ (4%) and static ProtUE *m*_*S*_ (7%), while for the dynamic ProtUE *b*_*D*_ and stability *s*_*D*_, and for the static stability *s*_*S*_, variability was rather large with 51%, 60% and 41%, respectively. Generally, stability measures for dynamic and static approaches (*s*_*D*_ and *s*_*S*_) show larger variation than their corresponding ProtUE measures (*b*_*D*_, *m*_*S*_).

**Fig. 6:**
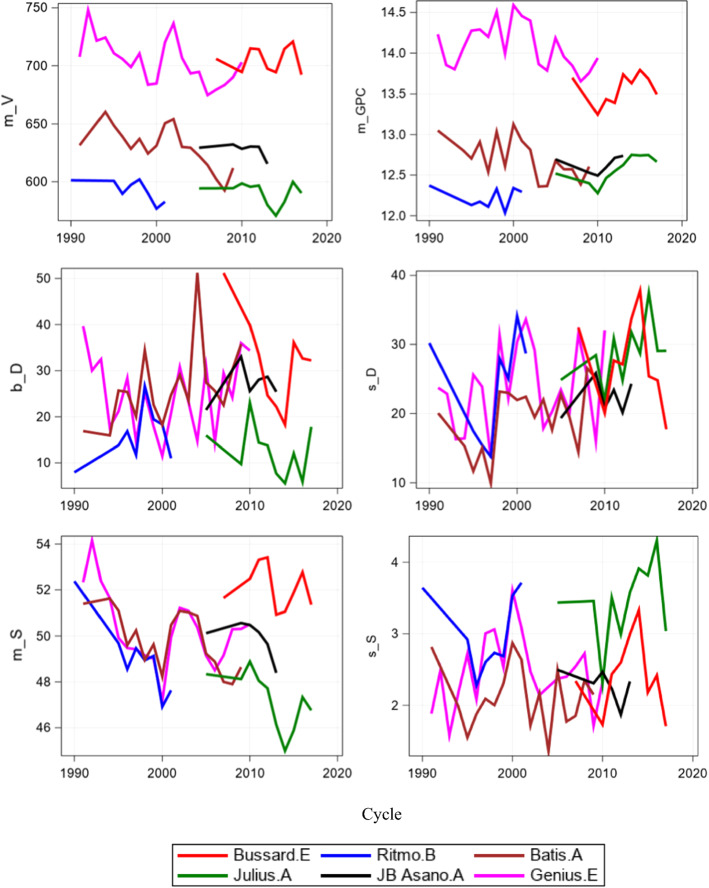
Profiles of variety × cycle parameters of dynamic and static ProtUE and stability for reference varieties. *m_V* cycle mean of loaf volume (*m*_*V*_); *m_GPC* cycle mean of grain protein content (*m*_*GPC*_); *b_D* Dynamic ProtUE (*b*_*D*_); *s_D* Dynamic stability (*s*_*D*_); *m_S* Static ProtUE (*m*_*S*_); *s_S* Static stability (*s*_*S*_)

Besides a very high heritability for the *m*_*V*_ and *m*_*GPC*_, both with *h*^2^ = 96%, Fig. 5 depicts that heritability of ProtUE and stability of the static approach was considerably higher than for the measures of the dynamic approach. Heritability of the static ProtUE *m*_*S*_ (*h*^2^ = 92%) reached nearly that of *m*_*V*_ and *m*_*GPC*_. We visualized the effect of high and low heritability in Fig. 6 by plotting variety × cycle profiles of 6 reference varieties for individual measures against successive testing cycles. Profiles of varieties with a widespread and parallel pattern indicated high heritability, as shown for *m*_*V*_, while narrow profiles with more overlapping and crossing profiles indicated lower heritability, as was the case for the dynamic ProtUE *b*_*D*_ and stability *s*_*D*_. Profiles of varieties for the static ProtUE (*m*_*S*_) in Fig. 6 show a more parallel and less dispersed pattern than for the dynamic ProtUE (*b*_*D*_) *H* Heritability.

### Genotypic correlation of dynamic and static ProtUE measures

**Fig. 7:**
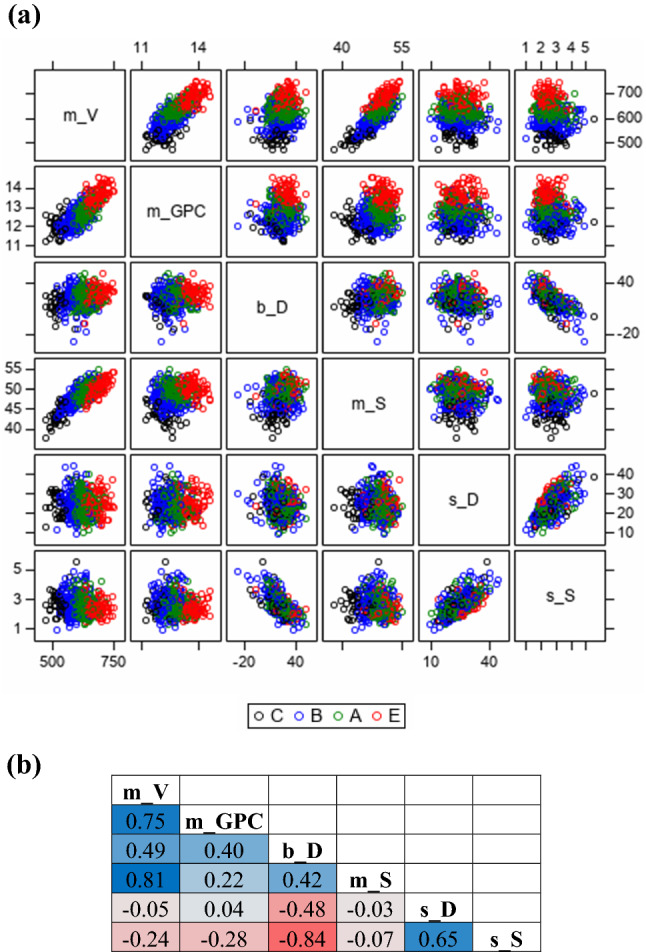
**a** Correlation matrix of variety × cycle measures indicating quality groups with grade C, B, A and E. **b** Genotypic correlation coefficients for variety × cycle measures. The blue-grey-red heat map indicates the strength of correlation from positive (blue) to negative (red) correlation coefficients. *m_V* cycle mean of loaf volume (V); m_*GPC* cycle mean of grain protein content (GPC); *b_D* Dynamic ProtUE (*b*_*D*_); *s_D* Dynamic stability (*s*_*D*_); *m_S* Static ProtUE (*m*_*S*_); *s_S* Static stability (*s*_*S*_)

Association of 6 variety × cycle measures is shown in the symmetric matrix plot of Fig. 7a with differently colored quality groups. The association of cycle means for V (*m*_*V*_) and GPC (*m*_*GPC*_) with other measures showed clearly separated clusters for quality groups C, B, A and E. However, this clustering into quality groups was weak or absent for the associations between dynamic and static ProtUE and stability. The dispersion of clusters was different: clusters for E-grade varieties are less dispersed than for B-grade varieties as shown for example between *s*_*D*_ and *s*_*S*_ in Fig. 7a. Genotypic correlation coefficients are given in Fig. 7b. Strong positive genotypic correlations were found for *m*_*V*_ with *m*_*GPC*_ (*r* = 0.75) and with *m*_*S*_ (*r* = 0.81), and between the dynamic ProtUE (*s*_*D*_) and static stability (*s*_*S*_) with *r* = 0.65. The dynamic ProtUE (*b*_*D*_) and the static stability (*s*_*S*_) were strongly negatively correlated with *r* =−0.84. This association means that with higher dynamic ProtUE the stability of V/GPC ratio is increasing. The other correlations were weak to moderate in the range of *r* =  − 0.48 (*b*_*D*_, *s*_*D*_) and *r* = 0.49 (*m*_*V*_, *b*_*D*_).

## Discussion

### Genotypic and environmental variation and correlation

The genotypic and environmental variation of V, GPC and V/GPC, quantified in terms of stability using Eq. (1) and depicted in Fig. 1, is important to the milling and baking industry, whose processing technology requires consistent raw materials. For V, the most important trait of baking quality, environmental variation accounted for only 24% because V is more related to protein quality, reflected in glutenin allelic composition and concentration which is most variety-dependent (e.g., Graybosch et al. 1996; Bilgin et al. 2015; Johannson et al. 2020). Compared to V, GPC was very sensitive to environments (57%), especially the influence of locations. The influence of years was much larger for GPC than for V, which agrees with results from, e.g., Rozbicki et al. (2015) and Laidig et al. (2017). The strong influence of environmental factors on the variation of GPC is mostly governed by the nitrogen availability and variety development time (Hafeez et al. 2013; Johannson et al. 2020).

Figure 1 shows that the relative magnitude of variance components for individual effects of V/GPC were about between V and GPC, however, with some shifts influenced by strength of the correlation between effects of V and GPC. We should consider that the V–GPC relation is the ratio of two traits, both being subject to more or less large environmental conditions as became apparent by Fig. 1, but with well-known positive association between V and GPC. Generally, the variance of the ratio between two random variables is also dependent on the magnitude of their correlation. Equation (9) shows that the variance decreases with increasing positive correlation. For example, if V is strongly positively correlated with GPC, then the variance of V/GPC should be smaller compared to the variance of V/GPC when V and GPC are weak correlated. For V/GPC, Fig. 2 shows that the random effects for year Y were moderately correlated between V and GPC with *r* = 0.44 and Fig. 1 indicates that the variance component of Y was 10% of the total variance, which was larger than for V (3%) and GPC (2%) due to a moderate positive correlation of Y between V and GPC. The variance component for location L of V/GPC was low (3%), while the corresponding variation for V (4%) and GPC (11%) was higher because the random effects of V and GPC were very strongly correlated (*r* = 0.90). For the same reason, the residual variation for V/GPC (20%) was about twice as large as for V and GPC with *r* = 0.29. Overall, the strong to very strong association of random effects for location and year × location mitigated the relative environmental variation of V/GPC compared to GPC, while the variation of the genotype × environment increased.

### Prediction of loaf volume by protein content

V increased with increasing GPC in a curvilinear relationship as shown in Fig. 3 for all quality groups. However, the comparison of the V–GPC relation in 1990 with that in 2015 indicated that for all quality groups lower values for V were predicted in 2015 in the range of 46 and 53 ml given the same GPC. This linearly decreasing time trend was significant for all quality groups (Table 1). Results reported in the literature (Cox et al. 1989; Uzik et al. 2009; Hartl et al. 2011; Laidig et al. 2017) generally agreed that considerable gain in grain yield was achieved during the last decades, but are inconsistent as to whether or not significant progress in baking quality has been made. Pronin et al. (2020) analyzed grain yield, GPC and gluten composition of 60 German winter wheat varieties registered between 1891 and 2010 grown at one location in 3 years. While yield increased over time, GPC, gliadin contents and gliadin/glutenin ratio showed a decreasing trend, glutenin contents increased, but there were no changes in albumin/globulin and gluten contents. They suggested that the protein composition may have changed as a result of breeding practices targeting wheat with elastic dough properties. From this, it can be concluded, even though it was not explicitly investigated in this study, that breeding contributed to an increase of V despite a decreasing trend of GPC. Also, Hartl et al. (2011) tested 94 German winter wheat cultivars released since 1961 at 7 environments in Bavaria over 2 years. They concluded that for widely used cultivars an increase of 20% for grain yield together with an increase of V of about 50 ml could be attested. This differs clearly from our results. We attribute this difference to the fact that there has been a relative increase in GPC quality and V due to breeding, but this improvement has not been able to compensate for the decreasing GPC. This is supported by Laidig et al. (2017), where we found for VCU trials a large significant genetic gain in grain yield, a significant decline in GPC and a moderate decline in V. Moreover, Hartl et al. (2011) carried out field trials over 2 years with a historical set of 94 varieties with quality grades B to E, whereas our study covered 355 released varieties including all quality grades from C to E grown in 32 years over a wide range of environments and cultivation conditions across Germany.

The steepness of the curvilinear increase in Fig. 3 increased gradually from C- to E-group, which showed that varieties in the E-group had a higher ProtUE. This further indicated that with increasing GPC-level a decreasing amount of V has been achieved, in other words, the increase of GPC from 12–13% yields higher V than from 13–14%. This curvilinear relationship between V and GPC was already reported by Bailey and Sherwood (1926), who concluded that “Increase in loaf volume with each unit increment of increase in protein content diminishes with increasing percentages of protein”.

It is well known that GPC increases with increasing N fertilization up to a maximum and then dropping. Borghi (1999) stated that “GPC of wheat increases with increasing N fertilization and usually reaches a peak at a N fertilisation level above that needed to achieve maximum yield. After this peak the protein quality decreases with increasing GPC because the extra N accumulated in the grain is represented by gliadins or non-protein nitrogen, which downgrades the baking quality”. Weegels et al. (1996) reported that curvilinear relationship between V and GPC may be explained by changes in protein composition and quality in different quality groups and varieties.

The curves of the V–GPC relation (Fig. 3) can be considered as the average across all varieties in individual quality groups. Some varieties, however, may show a different V–GPC relation. Gabriel et al. (2017) evaluated the V–GPC relation of 180 winter wheat genotypes from more than 1000 grain samples grown under a wide range of N fertilization levels (0–220 kg N) and GPC (6.7–17.1%). They found that most genotypes showed a curvilinear relationship, but some genotypes also displayed a linear relationship. Altogether, their results confirmed that the ProtUE of varieties differed along GPC. Overall, the regression functions showed that (i) the average ProtUE decreased from 1988 to 2019, (ii) ProtUE increased curvilinear from C- to E-grade varieties, and (iii) the gain of V per unit GPC decreases with increasing GPC.

### Variance components, heritability and genotypic correlation of dynamic and static ProtUE measures

The core objective of this study was to evaluate measures of ProtUE and their environmental stability for a potential use in variety registration as additional criteria to assess a variety’s VCU. The motivation was the demand of new varieties with high and stable baking quality in face of reduced N application requirements and changing climate with more volatile growing conditions. However, baking tests in wheat registration trials are expensive and laborious. Therefore, only a limited number of samples from 3 years and at most 8 environments per year were available to calculate performance measures. Our estimates of variance components (Fig. 5) showed that heritability of the dynamic ProtUE and stability was low (*h*^2^ = 43%, *h*^2^ = 31%, respectively), whereas heritability for the static ProtUE was considerably higher (*h*^2^ = 92%, *h*^2^ = 51%, respectively).

To the best of our knowledge, no other study on the V–GPC relationship evaluating stability and usability for variety selection is available. All studies on stability of yield and baking quality are based on individual traits. Results for yield were reported by Heine and Weber (1982) from winter wheat and maize registration trials, and by Becker and Leon (1988) for wheat, barley and oat post-registration trials. Their results showed no sufficient potential to predict stability of varieties given the typical number of environments in such trials. Further, Sneller et al. (1997) published results on the repeatability of yield stability of regression coefficients in soybean. They considered repeatability of measures based on variety rankings between trial series as having a low potential for selection of stability. Ortiz et al. (2001) studied the heritability of genotype-by-environment stability measures for grain yield in bread wheat based on diallel crosses derived from 8 lines grown at 3 locations in Uganda and grown in several seasons. The highest heritability was found for the coefficient of phenotypic variation for yield with *h*^2^ = 0.52, whereas the heritability for regression coefficient *b* and coefficient of determination *R*^2^ were low (*h*^2^ = 0.15 and 0.10, respectively). Robert (2002) compared various stability measures for yield and quality traits in bread wheat and evaluated their repeatability by using rank correlations. They concluded: “Whatever the trait and statistic envisaged, stability is poorly repeatable and its evaluation requires several years and a large number of locations per year to minimize sampling and environmental effects.” Overall, reported results on the usability of stability measures based on single traits only were not promising.

Selection for dynamic and static ProtUE and stability should not counter-act the selection for high V and GPC (i.e., the selection of varieties with high ProtUE and stability does not risk retention of varieties with low V (Robert 2002)). Figure 7a and b demonstrates the positive genotypic correlations between cycle means V and GPC (*r* = 0.75), and between V and dynamic ProtUE *(r* = 0.49) and stability (*r* = 0.81). This indicates that selection for high dynamic and static ProtUE is likely to show high cycle means for V. Further, dynamic stability was nearly independent from cycle means for V, i.e., does not counteract with selection for high V. Selection for high static stability was only weakly associated with high V (*r* = −0.24), this demonstrated that selection for high static stability supports selection for higher V and vice versa. In summary, selection of varieties with high static ProtUE and stability provided a stronger support of selecting varieties with high V as compared to dynamic ProtUE and stability.

## Conclusions

New varieties with a high potential of grain ProtUE are very important with respect to the goal of agricultural policies in the European Union, including Germany, to reduce the surplus of nitrogen fertilizer in certain areas where nitrogen leaching is large. Additionally, ProtUE needs to be very stable across environments in face of progressing climate change with more volatile growing conditions from location to location and year to year.

We evaluated a new approach considering the stability of the V–GPC relationship of baking quality for winter wheat instead of considering individual traits only. Genotypic and environmental variation of V/GPC was high and intermediate between that of V and GPC. The variance for locations and location × year interaction had a very strong impact on variation of V and GPC. Prediction of V by GPC for quality groups C, B, A and E showed a curvilinear relationship where the predicted V-level and the steepness increased from C- to E-grade varieties indicating an increasing ProtUE of quality groups. However, ProtUE showed a curvilinear decreasing trend from 1988 to 2019, which means that on the average less V was predicted in recent years per 1% GPC.

Heritability of dynamic ProtUE and stability was low compared to V, GPC and V/GPC and consequently their repeatability from cycle to cycle. A very high heritability was found for the static ProtUE, close to that of cycle means for V and GPC while the static stability was of moderate magnitude. Selection for high dynamic and static ProtUE showed no counter-selection for high V. In fact, static ProtUE is highly correlated with the cycle mean of V. Further, dynamic and static stability measures were not or only weak negatively correlated with the cycle means of V and GPC. Static stability was even negatively weak associated with higher cycle means for V indicating that selection for high stability will likely be associated with high V.

Our results showed that static ProtUE is highly repeatable and stability is moderately repeatable and should be considered as additional efficient quality-related descriptors in breeding and testing new winter wheat varieties with high potential for ProtUE and its stability.

## Supplementary information

Below is the link to the electronic supplementary material.

Electronic supplementary material SM Estimation of correlations between random effects of two variables based on a univariate approach.Supplementary file1 (DOCX 17 KB)

## Data Availability

The datasets analyzed during the current study are available from the corresponding author on reasonable request.
