# Supplementary material for: Protein use efficiency and stability of baking quality in winter wheat based on the relation of loaf volume and grain protein content
Source: Theor Appl Genet. 2022 Jan 28;135(4):1331–43. doi: 10.1007/s00122-022-04034-x (PMC9033720; doi:10.1007/s00122-022-04034-x)
Supplement: Electronic supplementary material — SM Estimation of correlations between random effects of two variables based on a univariate approach.Supplementary file1 (DOCX 17 KB) [file 122_2022_4034_MOESM1_ESM.docx]

**Supplementary Material SM**

*Estimation of correlations between random effects of two variables based on a univariate approach*

A bivariate model was assumed with given random effects. To estimate the correlation between random effects $H_{i}^{(p)}$ and $H_{i}^{(q)}$for variables (*p*) and (*q*)*,* where, for example, $H_{i}^{(p)}$ and $H_{i}^{(q)}$could represent the dynamic PUE and dynamic stability *b_D_* and *s_D_*, respectively. However, for computational reasons we used a univariate approach from which correlations for pairs of variables can be inferred (Piepho et al. 2014):

1. Calculate variance components according to the random effects of given model (Eq. (12)) for variable (*p*) and (*q*) and for the difference between both variables.

2. Compute covariances between the random effects *H_i_* of measure (*p*) and (*q*)

from variance components obtained from univariate models by using the equation

$var{(H_{i}^{\left( p \right)}-H_{i}^{(q)})}_{i}$= var ($H_{i}^{(p)}$) + var ($H_{i}^{(q)}$) 2cov ($H_{i}^{(p)},H_{i}^{(q)})$ [1]

cov($H_{i}^{(p)},H_{i}^{(q)})=\frac{var(H_{i}^{\left( p \right)})+var(H_{i}^{\left( q \right)})var(H_{i}^{\left( p \right)}-H_{i}^{(q)})}{2}$ [2]

(3) Use variances from Eq. [1] and covariance from Eq. [2] to calculate the correlation coefficients *r* for random effects.

**Reference**

Piepho, H.P., Mueller, B.U., Jansen, C., 2014. Analysis of a complex trait with missing data on the component traits. Communications in Biometry and Crop Science 9, 26-40.
